# Feasibility of Magnetic Resonance‐Guided High‐Intensity‐Focused Ultrasound (MRgHIFU) Ablation of Stump Neuromas for the Relief of Chronic Postamputation Neuropathic Pain

**DOI:** 10.1002/jum.16026

**Published:** 2022-05-28

**Authors:** Alicia Nachtigal, Ronen Cozakov, Anat Grinfeld, May Haddad, Elon Eisenberg

**Affiliations:** ^1^ Department of Radiology Hillel‐Yaffe Medical Center Hadera Israel; ^2^ Institute of Pain Medicine Rambam Health Care Campus Haifa Israel; ^3^ Department of Radiology Rambam Health Care Campus Haifa Israel; ^4^ Rappaport Faculty of Medicine Technion, Israel Institute of Technology Haifa Israel

**Keywords:** nerve block, neuropathic pain, phantom pain, residual limb pain, thermal ablation

## Abstract

Up to 70% of limb amputees develop chronic postamputation neuropathic pain (CPANP) which includes phantom pain and residual limb neuropathic pain due to neuroma formation. CPANP often requires invasive procedures aimed at neuroma ablation. Five amputees received 6 noninvasive magnetic resonance‐guided high‐intensity‐focused ultrasound MRgHIFU treatments ExAblate®, Insightec, Tirat‐Carmel, Israel). Although ablative temperature (>65°C) at the neuroma was reached in only 1 patient, pain intensity dropped from 5.7 at baseline to 4.3 and back to 5.6 at 3 and 6 month follow‐up. Post‐treatment bone necrosis was demonstrated in 1 patient. Although no firm conclusion about the effectiveness of MRgHIFU for CPANP could be drawn, further studies are warranted.

**Abbreviations**
CPANP: chronic postamputation neuropathic pain
HIFU: high‐intensity‐focused ultrasound
MR: magnetic resonance
MRgHIFU: magnetic resonance‐guided high‐intensity‐focused ultrasound
MRI: magnetic resonance imaging
NPS: numerical pain scale
US: ultrasound

In 2005, 1.6 million people were estimated to be living with limb loss in the United States^1^ and the number is projected to double by the year 2050.^2^ Ninety‐five percent of patients, who undergo limb amputation report experiencing some amputation‐related pain and 70%–80% develop chronic postamputation neuropathic pain (CPANP) in the affected limb, which involves the missing limb (phantom), the residual limb neuropathic pain, or both.^3^ Although the underlying mechanisms are not fully clarified, growing evidence suggests that changes in afferent nerves, including the formation of neuromas, leading to increased ectopic afferent input, play an important role in the pathogenesis of CPANP.^4^, ^5^ CPANP is often persistent, resistant to medical treatments, and leads to considerable disability in many patients.^6^ Therefore, invasive treatment modalities aimed at neuroma ablation, such as neurolytic injections, radiofrequency therapy, or even surgical resection of neuromas, are often required, although the literature regarding their efficacy and safety is often scarce.^7^, ^8^, ^9^, ^10^Magnetic resonance‐guided high‐intensity‐focused ultrasound (MRgHIFU) is a noninvasive procedure that combines 2 technologies: 1) high‐intensity‐focused ultrasound (HIFU) waves, which are converted to thermal energy at a precise focal point, thus rising tissue temperature in the target area to approximately 65°C–85°C and resulting in irreversible thermal ablation of the treated tissue; 2) three‐dimensional magnetic resonance (MR) imaging of the focal point (neuroma in our case), which allows anatomical planning of the treatment by positioning the ultrasound (US) bean to optimize the effect on the target tissue while avoiding damage to surrounding tissue. The magnetic resonance imaging (MRI) also provides real‐time thermal imaging to ensure sufficient temperature rise of the target tissue but not of surrounding tissues.^11^, ^12^

MRgHIFU is utilized for painful metastatic bone tumors and osteid osteoma in the extremities. It has been studied in other painful conditions, for ablating small, localized sensory nerves in patients with knee osteoarthritis and facet joint arthropathy. It is also used for nonpainful condition including tremor (essential and tremor‐dominant Parkinson's disease), uterine fibroids, and ablation of prostate tissue (for review^13^).

The preset, first in human feasibility study was aimed to provide initial information on the effectiveness (pain reduction and safety) of MRgHIFU in limb amputees with CPANP.

## Case Descriptions

The study was approved by Rambam Health Care Campus Ethics Committee (RMB 0180‐16). Patients with CPANP who met the following inclusion criteria were recruited to the study: men and women aged 18 and older, suffering from CPANP for at least 3 months; able and willing to give informed consent; average pain intensity (when present) ≥4 on a 0–10 numerical pain scale (NPS) during 3 days prior to enrollment, irrespective of medication used; not more than 3 targeted neuromas, clearly visible by noncontrast MRI and located deeper than 10 mm from the skin; and temporary pain relief following a local anesthetics injection to the targeted neuroma(s) or to the relevant peripheral nerve(s). Patients were excluded from the study if they had: any acute or unstable medical condition that was expected to hinder them from completing this study; standard contraindications for contrast MRI or for sedation; and unwillingness to undergo the procedure under prolonged sedation (approximately 3 hours).

Patients were prescreened by a comprehensive medical and pain history, recording of current symptoms and a general physical and a detailed neurological examination of the stump. After signing an informed consent form, eligible patients were referred to a contrast MRI of the stump (unless a MRI scan was performed within 6 months prior to enrollment in the study). Subsequently, an US‐guided local anesthetic injection was performed to either the identified neuroma(s) or the involved nerve(s), proximal to the neuroma(s). A 50% pain relief was considered a successful injection.

The procedure was mostly performed on an outpatient basis. Patients received sedation (midazolam and fentanyl) with heart rate and pO_2_ monitoring under the care of a certified anesthesiologist throughout the procedure. Nerve block was added to 1 patient (001) prior to the procedure. Treatment was performed using the ExAblate MRgHIFU system (InSightec, Tirat Carmel, Israel). Patients were positioned on the MRgHIFU table with the targeted neuroma centered above the ultrasound transducer. The treated limb was fixated to the ExAblate table with adhesive plasters. Position, clear ultrasound pathway, and target neuroma's location and size were verified by MRI (T2‐ and T1‐weighted sequences in all 3 orientations).

**Figure 1:**
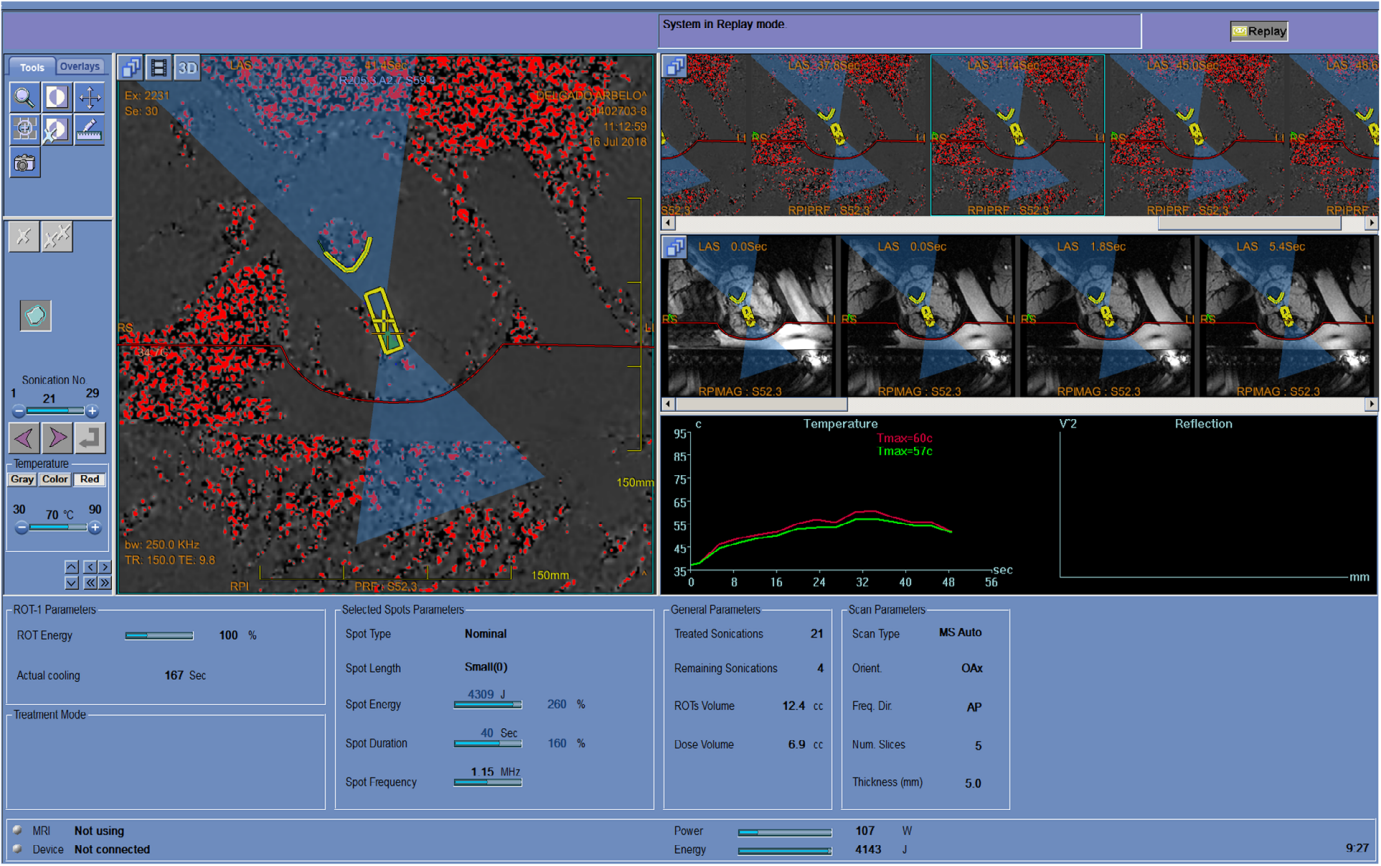
A representative screenshot from the ExAblate User Interphase BONES application during treatment.

Images were loaded to the ExAblate workstation, and the target area was marked. A patient‐specific treatment plan was generated covering the targeted neuroma optimizing the number of sonications, location, and energy levels to avoid damage to surrounding tissue. After verifying correct positioning using low‐energy sub‐therapeutic sonications, treatment began at full energy, attempting to reach ablation temperatures of 65°C–85°C. During each sonication, real‐time MR thermometry and anatomical images were acquired (Figure 1, Table 2). At treatment completion, T1‐weighted fat‐saturated contrast‐enhanced MR images were acquired.

Change from baseline in the average daily pain intensity (when present), measured for 3 consecutive days on the NPS was considered the primary outcome measure. Secondary endpoints included changes from baseline of the followings: 1) maximum daily pain intensity, measured for 3 consecutive days, on the NPS (0–10); 2) minimum daily pain intensity, measured for 3 consecutive days, on the NPS (0–10); 3) Short‐Form McGill Pain Questionnaire score (0–45); 4) Pittsburgh Sleep Quality Index (0–21); 5) quality of life measured by the EQ‐5D questionnaire (0–15); 6) Brief Pain Inventory (0–70); 7) MRI findings in the treated neuroma(s); and 8) treatment complications.

Telephone follow‐up visits were completed at 1 and 3 days, and 2 weeks post‐treatment and clinic visits at 1 week and 1, 3, and 6 months post‐treatment. A follow‐up MRI to assess the effects on the treated neuroma(s) was performed 4 months after treatment.

Twenty‐one amputees were prescreened between April 2017 and October 2018. Thirteen of them were excluded from further procedures for the following reasons: reluctant to undergo the procedure,^5^ pain intensity <4,^2^ high risk for sedation, active cancer, cardiac pacemaker, metal shrapnel, no evidence for neuroma per recent MRI and a very short leg stump (1 each). Eight patients signed an informed consent form. However, 3 of them did not receive treatment due to the following reasons: withdrew consent, infection in opposite other limb and absence of neuroma per study MRI (1 each). Hence, 5 patients received 6 HIFU treatments (1 patient received 2 consecutive treatments), all for lower limb neuromas. Table 1 presents the demographic characteristics of the 5 treated patients.

**Table 1:** Demographic and Pain Characteristics of the Treated Patients

Patient ID	p001	p005	p010	p012	p018
Age	66	42	68	48	84
Sex	Female	Male	Male	Male	Female
Time since amputation (months)	60	19	96	7	22
Amputated limb	Left leg	Left leg	Right leg	Right leg	Left leg
Reason for amputation	Ischemia	Trauma	Trauma	Infection	Ischemia
Type of pain	Phantom and stump	Phantom	Stump	Phantom and stump	Phantom
Pain pattern	Constant	Constant	Attacks	Constant	Attacks
Average pain intensity (0–10)	5.0	6.6	5.6	7.0	10.0

Altogether, 6 neuromas were treated. In 4 patients, a single tibial neuroma was treated. One patient (p001) had 3 neuromas (tibial, peroneal, and sural); the tibial neuroma was treated twice and the peroneal once. Due to safety hazards, full energy treatment of the sural neuroma was not initiated. The number of sonications ranged from 7 to 29. Despite of repeated sonications over relatively long treatment times (between 3:00 and 4:20 hours), ablation temperatures of 65°C was reached in only 4 of 107 administered sonications, all in 1 patient (p010). Overheating off adjacent tissues, mainly bone, was the reason for avoiding higher sonication energy levels. Table 2 summarizes the location and size of the neuromas and the main treatment parameters (number of sonications, maximal temperature reached at the target zone, and duration of treatment) and Figure 1 shows the MRgFUS system screen at the time of treatment. More details on treatment parameters of the 5 patients can be found in Table S1.

**Table 2:** Summary of Treated Neuromas

Patient ID	p001	p005	p010	p012	p018
No. of neuromas	3	1	1	1	1
Site (nerve) and size (cm)	Tibial 1.3 × 1.1 × 0.8 Sural 0.4 × 0.5 × 1.3 Peroneal 0.4 × 0.7 × 1.0	Tibial nerve: 3.4 × 0.8 × 1.3	Tibial nerve 1.0 × 1.0 × 2.3	Sciatic nerve 1.0 × 2.0 × 7.0	Tibial nerve 1.5 × 1.3 × 1.1
Number of treated neuromas	2 (Tibial and peroneal—1st treatment; tibial—2nd treatment); sural neuroma—not treated	1	1	1	1
Sonications (N)^a^	7 (1st treatment) 13 (2nd treatment)	12	24	29	22
Max temperature (°C)^b^	54 (1st treatment) 55 (2nd treatment)	60	78	61	56
Treatment time (hours)^c^	3:25 (1st treatment) 4:10 (2nd treatment)	3:00	4:20	3:55	3:25

^a^ Number of sonications administered to each neuroma, attempting to reach the ablation temperature. Sonications may differ from each other in the energy used, angle of sonication, and size and location of the treated zone.

^b^ Highest temperature reached at the target zone during all sonifications of each specific neuroma.

^c^ Total duration of treatment.

**Figure 2:**
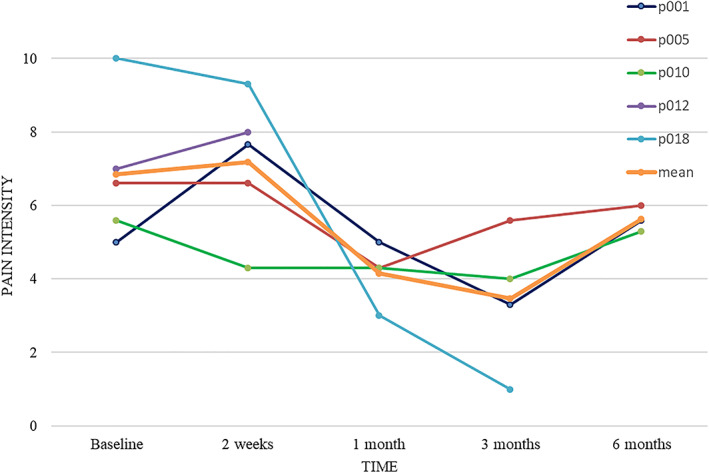
Average daily pain intensity before and after treatment. Pain intensity was measured on a 0–10 numerical pain scale for 3 consecutive days at each time point.

Three of the 5 treated patients (p001, p005, and p010) completed the entire 6‐month follow‐up. An additional patient (p012) completed the 2‐week telephone follow‐up but was unwilling to comply with the scheduled follow‐up clinic visits due to worsening of his phantom pain following the procedure. Another patient (p018) passed away from lung cancer and missed her 6‐month follow‐up visit. Figure 2 displays the average daily pain intensity (study primary outcome), reported by the treated patients before and after treatment. Overall, their average pain dropped from a mean of 7.2 at baseline (all 5 patients) to a minimum of 3.5 at 3‐month (based on reports of 4 patients) and increased to 5.6 at the 6‐month follow‐up time point (based on 3 patients). Pain reports from the 3 patients who competed the entire follow‐up period were 5.7 at baseline and 6.2, 4.5, 4.3, and back to 5.6 at 2‐week, 1, 3, and 6 month, respectively. Detailed outcomes of each individual patient can be found in Appendix S1 and in Tables S2–S6.

Follow‐up MRI scans were performed 1–6 months after treatment in all 5 treated patients, and were compared to the baseline findings in term of neuroma size, signal, and enhancement with gadolinium. No changes in any of these parameters were noted in all treated neuromas (Table S7).

Although all procedure lasted considerable time (hours) and required prolonged sedation, no safety hazards were recorded in any of the patients. Patients' safety or comfort did not require stopping any procedure prematurely. One patient (p012) reported aggravation in his pain 2 weeks after treatment. He declined further follow‐up clinic visits but made his follow‐up MRI scan available for our review. In another patient (p018) an MRI scan 1 month after the procedure showed findings consistent with post‐treatment bone and soft tissue infarction in the posterolateral facet of the tibia and the adjacent soft tissue, close to the stump neuroma. Nonetheless, the patient reported dramatic pain relief at that time. Unfortunately, she passed away so no additional follow‐up MRI was performed.

## Discussion

The aim of the present feasibility study was to provide initial information on the effectiveness (pain reduction) and safety of MRgHIFU treatment directed at ablating stump neuromas in amputees with CPANP. As often happens in studies with well‐defined inclusion criteria, only 5 of 21 prescreened amputees were eventually treated. Of them, only 3 completed the 6‐month follow‐up period. Nonetheless, with the exception of 1 patient, in whom pain escalated soon after the procedure, at least some pain relief was noted by the others for at least 3 months. Similar improvement was reported in additional, secondary outcome measures.

Due to previously mentioned technical challenges, ablative temperature was reached only in a small minority of administered sonifications. Thus, not surprisingly, no changes in the size, signal, or gadolinium enhancement could be detected in any of the treated neuromas. As such, the study team decided against performing further procedures with the ExAblate apparatus, even though pain intensity declined in 4 of the 5 treated patients. Whether the noted decline in pain intensity can still be attributed to the administered treatment is a valid question. A possible answer to this question may come from a different ablative technique—the radiofrequency. Several reports on the effect of ablative radiofrequency treatment (where target temperature far above 65°C has been reached) yielded positive results,^7^, ^8^, ^14^ congruently with to what we aimed to achieve with the MRgHIFU treatment. However, in at least 1 study in patients with Morton's neuroma, US‐guided *pulsed radiofrequency* treatment (with only a *sub‐ablative temperature* of ~42°C rather than true ablative temperature) was administered. Nonetheless, decrease in pain intensity was reported by 18 of 20 treated patients.^15^ Although the exact mechanisms by which sub‐ablative heat reduces neuroma pain are unknown, the results of Deniz et al,^15^ much alike ours, suggest that nondestructive thermal manipulations of neuromas may yield analgesic effects.

From the safety standpoint, no serious adverse events occurred in any of the patients. However, 2 safety concerns should be considered. First, the MRI findings suggesting post‐treatment bone and soft tissue infarction in patient 012. Although this finding was unlikely of clinical significance since the patient reported dramatic pain relief for 3‐month following the procedure, the MRI findings still suggest that even under careful, real‐time temperature control, surrounding tissues can get affected. Second, the procedure required a long sedation time, which is not entirely free of potential hazards. Additionally, the cost of the procedure should be considered and compared to alternative ablating techniques, especially in light of the long and costly scan—and treating team—time required for the MRgHIFU procedure.

As already mentioned, the main treating challenge originated from the technical aspects of the MRgHIFU device (ExAblate), which did not allow reaching an ablative temperature at the target site in a safe manner in the majority of the sonifications. The leading reason for that is that in this device, the US beams came from a relatively narrow angle, below the sedated patient. This considerably limited the options of aiming the beam to the required target zones while assuring a clear ultrasound pathway. Additionally, the ExAblate device only allowed the use of a whole body MR coil rather than a limb coil. This reduced the scan resolution to a degree where it was at times difficult even for an experienced musculoskeletal radiologist to visualize the neuromas during the procedure. Conclusively, we suggest that a modified device, perhaps with a dome‐shaped transducer placed around the stump edge, compatible with a limb coil, will likely allow overcoming some of these technical challenges.

Taken together, no firm conclusion regarding the effectiveness and safety of MRgHIFU for CPANP can be drawn from this feasibility study. Nonetheless, the authors still believe that noninvasive HIFU techniques aimed at ablating neuromas and safely relieving CPANP should not abandoned. Further studies with properly adjusted technologies in a larger sample size should be carried on.

## Supporting information


**Appendix S1** Supporting Information.

**Appendix S2** Tables.
