# Supplementary material for: Feasibility of Magnetic Resonance‐Guided High‐Intensity‐Focused Ultrasound (MRgHIFU) Ablation of Stump Neuromas for the Relief of Chronic Postamputation Neuropathic Pain
Source: J Ultrasound Med. 2022 May 28;41(12):3119–24. doi: 10.1002/jum.16026 (PMC9796504; doi:10.1002/jum.16026)
Supplement: Supplementary file 1 — Appendix S1 Supporting Information. [file JUM-41-3119-s002.docx]

**Supplemental text file I**

***Description of the individual patients:***

**Patient 001**

A 66 years old female, generally healthy. Due to leg ischemia she underwent below knee amputation (left) in 2012. She subsequently developed severe constant residual limb and several daily attacks of phantom limb pain. A poor pain reduction and severe side effects to several anti-neuropathic pain medications and opioids were reported.

Her examination revealed allodynia to light touch at the tip and the back of the stump. A Tinel's sign above the peroneal nerve and to a lesser extent above the tibial nerve were evoked. Tapping over the tibial nerve resulted in electric currents along the leg down to the foot. Three neuromas were detected in a MRI scan (Tibial, Peroneal and Sural).

Following a sciatic block, a good pain relief was achieved and accordingly eligibility for the study treatment was established.

Two of the three neuromas (peroneal and tibial) were sonicated (total of 7 sonications; max area of interest (AOI) temp =61^0^C). The sural neuroma could not be approached and was not treated. Two weeks later she reported good pain relief of her residual limb pain but the phantom pain did not change. A second treatment was offered 4-weeks after the first one and the tibial neuroma was sonicated (13 sonications; max temperature at the area of interest (AOI) – 55^0^C). At the next follow up visit she reported further relief in the residual limb pain but worsening of the phantom pain. The maximal improvement was reported 3 months after the second treatments, with about 50% pain relief. However, at the 6-month visit, her pain returned almost to its baseline score.

The patient underwent MRI scan 4 months following treatment. Compared with the baseline MRI, no change was detected in size and signal but mild enhancement following gadolinium injection was noted in all three neuromas.

**Patient 005**

A 42 years old male, generally healthy. Due to a traumatic injury he underwent below left knee amputation in 2016. He suffered very severe constant phantom pain that poorly responded to several anti-neuropathic pain medications and opioids.
His examination revealed stump allodynia and positive Tinel's sign at the back of the stump (tibial nerve). The MRI scan demonstrated a long and twisted tibial neuroma only.

Following a tibial nerve block, good pain relief was achieved and the patient was found eligible for the treatment.

The tibial neuroma was sonicated (12 sonications; max AOI temp =82^0^C) and the patient completed a 6 months follow up.

The main improvement was reported at the 1-month follow-up visit, whereas at the 3 and 6 months pain gradually returned almost to its baseline value. The patient underwent MRI scan 4 months following treatment. Compared with the baseline MRI, no changes were detected in size, signal and positive contrast enhancement of the neuromas.

**Patient 010**

A 68 years old male, generally healthy. He underwent right below knee amputation as a result of a traumatic injury in 2002.

Since then, suffered residual limb pain that has not responded opioids and anti-neuropathic pain medications.

His examination revealed pressure hyperalgesia with no tactile allodynia over the stump. Tinel’s sign over the tibial nerve provoked his familiar pain but at a lower intensity than his spontaneous pain. MRI scan demonstrated a tibial neuroma.

Good pain was achieved with a peroneal nerve block. Hence, the patient was found eligible for the study treatment. The tibial neuroma was sonicated (23 sonications; max AOI temp =78^0^C).

An improvement was already reported at 2 weeks with maximal average pain reduction from 5.6 to 4.0 at three months. / At 56-month pain returned almost to its baseline value.

An MRI scan 4 months following treatment, showed no changes from baseline in neuroma’s size, signal and contrast.

**Patient 012**

A 48 years old male, with background of hypertension. He sustained an open fracture of his paroxysmal tibia in 2014, which was complicated with osteomyelitis, leading to above right knee amputation in 2017.

Since, suffered very constant residual limb pain and four phantom pain attacks a day. His pain was not controlled with anti-neuropathic pain medications and high opioid dose and medical cannabis. His examination revealed dynamic tactile allodynia around the stump tip. The MRI scan demonstrated a sciatic neuroma. Sciatic nerve block relived his pain temporarily and FUS treatment was given (29 sonications; max AOI temp =60^0^C).

The patient was seen only at the two-week follow-up and reported that his pain worsened, especially an in phantom pain frequency. He then decided not to show up for further follow-up visits. The only exception was and follow-up MRI study, performed 4 months following treatment, which failed to show changes from baseline in size, signal and contrast of the neuroma.

**Patient 018**

An 84 years old female, with background of hypertension, peripheral vascular disease and hypo-thyroidism. Due to an ischemia in her left leg, she underwent below knee amputation in 2015. Two years post amputation, she developed phantom pain, which typically appeared 15 minutes after wearing her prosthesis. MRI revealed a neuroma of the tibial nerve. Good pain relief following a nerve block confirmed treatment eligibility.

The tibial neuroma was sonicated. (22 sonications; max AOI temp =56^0^C) The patient completed the 2-week, 1 month and 3-month follow-up visits. She reported dramatic pain relief starting from the 1-month follow up. Notably, approximately at the same time she received a new prosthesis, which might have also contributed to her pain relief.

Due to a non-related illness (lung cancer) she could not complete the follow up visits.

An MRI scan one months following treatment showed no changes in size, signal and contrast of the neuroma. However, a round area involving the posterolateral facet of the tibia and the adjacent soft tissue was observed close to the stump neuroma, enhanced after Gadolinium administration, most probably represented a post-treatment bone and soft tissue infarction.
