# Supplementary material for: Feasibility of Magnetic Resonance‐Guided High‐Intensity‐Focused Ultrasound (MRgHIFU) Ablation of Stump Neuromas for the Relief of Chronic Postamputation Neuropathic Pain
Source: J Ultrasound Med. 2022 May 28;41(12):3119–24. doi: 10.1002/jum.16026 (PMC9796504; doi:10.1002/jum.16026)
Supplement: Supplementary file 2 — Appendix S2 Tables. [file JUM-41-3119-s001.docx]

| **Supplemental Table 1: Treatment parameters** | | | | | | | | |
| --- | --- | --- | --- | --- | --- | --- | --- | --- |
| Treated patient | Number of sonications | Sonication Plane | Total Energy [Jouel] | Power [Watt] | Duration [Sec] | Comments | Max temp  [^0^C]* | Total time in scanner |
| p001  First Treatment | 1 | Axial | 90 | 19 | 5 | Sonication aborted | 41 | 3h 25m |
|  | 2 | Axial | 436 | 18 | 24 |  | 41 |  |
|  | 3 | Axial | 437 | 18 | 24 |  | 41 |  |
|  | 4 | Axial | 521 | 21 | 25 |  | 41 |  |
|  | 5 | Axial | 708 | 29 | 24 |  | 53 |  |
|  | 6 | Axial | 405 | 17 | 24 |  | 42 |  |
|  | 7 | Axial | 479 | 20 | 24 |  | 54 |  |
| p001  Second Treatment | 1 | Coronal | 761 | 31 | 25 |  | 51 | 4h 10m |
|  | 2 | Axial | 1181 | 49 | 24 |  | 45 |  |
|  | 3 | Axial | 1512 | 63 | 24 |  | 52 |  |
|  | 4 | Axial | 756 | 49 | 15 | Sonication aborted | 45 |  |
|  | 5 | Coronal | 1162 | 48 | 24 |  | 52 |  |
|  | 6 | Coronal | 1543 | 48 | 32 |  | 42 |  |
|  | 7 | Coronal | 1538 | 46 | 33 |  | 54 |  |
|  | 8 | Axial | 1546 | 46 | 34 |  | 55 |  |
|  | 9 | Axial | 1686 | 49 | 34 |  | 49 |  |
|  | 10 | Axial | 1649 | 48 | 34 |  | 53 |  |
|  | 11 | Axial | 1763 | 48 | 37 |  | 49 |  |
|  | 12 | Axial | 1749 | 48 | 36 |  | 54 |  |
|  | 13 | Axial | 1747 | 48 | 36 |  | 52 |  |
| p005 | 1 | Coronal | 181 | 22 | 8 | Sonication aborted | 39 | 3h |
|  | 2 | Axial | 534 | 22 | 24 |  | 38 |  |
|  | 3 | Axial | 265 | 32 | 8 | Sonication aborted | 42 |  |
|  | 4 | Axial | 313 | 34 | 9 | Sonication aborted | 43 |  |
|  | 5 | Axial | 811 | 33 | 25 |  | 48 |  |
|  | 6 | Axial | 821 | 34 | 24 |  | 43 |  |
|  | 7 | Axial | 1278 | 43 | 30 |  | 60 |  |
|  | 8 | Axial | 1651 | 51 | 32 |  | 48 |  |
|  | 9 | Axial | 1558 | 53 | 29 |  | 52 |  |
|  | 10 | Axial | 1929 | 61 | 32 |  | 50 |  |
|  | 11 | Axial | 2001 | 68 | 29 |  | 49 |  |
|  | 12 | Axial | 2284 | 77 | 30 |  | 48 |  |
| p010 | 1 | Axial | 390 | 16 | 24 |  | 58 | 4h 20m |
|  | 2 | Axial | 483 | 25 | 19 |  | 55 |  |
|  | 3 | Axial | 483 | 25 | 19 |  | 78 |  |
|  | 4 | Axial | 413 | 21 | 20 |  | 75 |  |
|  | 5 | Axial | 721 | 37 | 19 |  | 71 |  |
|  | 6 | Axial | 390 | 16 | 24 |  | 54 |  |
|  | 7 | Axial | 457 | 23 | 20 |  | 45 |  |
|  | 8 | Axial | 534 | 27 | 20 |  | 48 |  |
|  | 9 | Axial | 693 | 36 | 19 |  | 48 |  |
|  | 10 | Axial | 830 | 43 | 19 |  | 51 |  |
|  | 11 | Axial | 1114 | 57 | 20 |  | 58 |  |
|  | 12 | Axial | 945 | 49 | 19 |  | 53 |  |
|  | 13 | Axial | 861 | 44 | 20 |  | 77 |  |
|  | 14 | Axial | 873 | 45 | 19 |  | 54 |  |
|  | 15 | Axial | 862 | 44 | 20 |  | 57 |  |
|  | 16 | Axial | 1067 | 55 | 19 |  | 56 |  |
|  | 17 | Axial | 864 | 44 | 20 |  | 67 |  |
|  | 18 | Axial | 864 | 44 | 20 |  | 58 |  |
|  | 19 | Axial | 868 | 44 | 20 |  | 62 |  |
|  | 20 | Axial | 867 | 44 | 20 |  | 74 |  |
|  | 21 | Axial | 868 | 44 | 20 |  | 57 |  |
|  | 22 | Axial | 859 | 44 | 20 |  | 48 |  |
|  | 23 | Axial | 947 | 49 | 19 |  | 51 |  |
|  | 24 | Axial | 950 | 49 | 19 |  | 61 |  |
| p012 | 1 | Axial | 599 | 25 | 24 |  | 41 | 3h 55m |
|  | 2 | Axial | 708 | 29 | 24 |  | 41 |  |
|  | 3 | Axial | 812 | 34 | 24 |  | 43 |  |
|  | 4 | Axial | 938 | 39 | 24 |  | 39 |  |
|  | 5 | Axial | 1342 | 69 | 19 |  | 44 |  |
|  | 6 | Axial | 1901 | 79 | 24 |  | 42 |  |
|  | 7 | Axial | 2355 | 121 | 19 |  | 46 |  |
|  | 8 | Axial | 2695 | 155 | 17 |  | 39 |  |
|  | 9 | Axial | 3031 | 196 | 15 |  | 45 |  |
|  | 10 | Axial | 3261 | 250 | 13 |  | 49 |  |
|  | 11 | Axial | 3238 | 249 | 13 |  | 61 |  |
|  | 12 | Axial | 3129 | 240 | 13 |  | 45 |  |
|  | 13 | Axial | 3262 | 289 | 11 |  | 54 |  |
|  | 14 | Axial | 3363 | 355 | 9 | Sonication aborted | 48 |  |
|  | 15 | Axial | 3373 | 357 | 9 | Sonication aborted | 52 |  |
|  | 16 | Axial | 3419 | 88 | 39 |  | 52 |  |
|  | 17 | Axial | 3428 | 88 | 39 |  | 43 |  |
|  | 18 | Axial | 3591 | 92 | 39 |  | 55 |  |
|  | 19 | Axial | 3597 | 92 | 39 |  | 49 |  |
|  | 20 | Axial | 3762 | 96 | 39 |  | 58 |  |
|  | 21 | Axial | 4143 | 107 | 39 |  | 57 |  |
|  | 22 | Axial | 4043 | 104 | 39 |  | 55 |  |
|  | 23 | Axial | 4172 | 107 | 39 |  | 53 |  |
|  | 24 | Axial | 4212 | 108 | 39 |  | 49 |  |
|  | 25 | Axial | 4278 | 110 | 39 |  | 60 |  |
|  | 26 | Axial | 4757 | 123 | 39 |  | 51 |  |
|  | 27 | Axial | 4403 | 114 | 39 |  | 46 |  |
|  | 28 | Axial | 4119 | 107 | 38 |  | 53 |  |
|  | 29 | Axial | 4247 | 110 | 39 |  | 49 |  |
| 018 | 1 | Axial | 850 | 35 | 24 |  | 44 | 3h 25m |
|  | 2 | Axial | 881 | 36 | 24 |  | 46 |  |
|  | 3 | Axial | 1103 | 46 | 24 |  | 43 |  |
|  | 4 | Axial | 972 | 40 | 24 |  | 56 |  |
|  | 5 | Axial | 1232 | 51 | 24 |  | 45 |  |
|  | 6 | Axial | 1474 | 61 | 24 |  | 52 |  |
|  | 7 | Axial | 1717 | 71 | 24 |  | 51 |  |
|  | 8 | Axial | 2004 | 83 | 24 |  | 47 |  |
|  | 9 | Axial | 2269 | 94 | 24 |  | 44 |  |
|  | 10 | Axial | 2519 | 105 | 24 |  | 47 |  |
|  | 11 | Axial | 2908 | 121 | 24 |  | 49 |  |
|  | 12 | Axial | 2163 | 90 | 24 |  | 54 |  |
|  | 13 | Axial | 2224 | 92 | 24 |  | 46 |  |
|  | 14 | Axial | 2576 | 106 | 24 |  | 44 |  |
|  | 15 | Axial | 1172 | 48 | 24 |  | 49 |  |
|  | 16 | Axial | 1643 | 68 | 24 |  | 50 |  |
|  | 17 | Axial | 1736 | 72 | 24 |  | 55 |  |
|  | 18 | Axial | 2099 | 87 | 24 |  | 49 |  |
|  | 19 | Axial | 2553 | 106 | 24 |  | 45 |  |
|  | 20 | Axial | 2530 | 105 | 24 |  | 51 |  |
|  | 21 | Axial | 2537 | 65 | 39 |  | 52 |  |
|  | 22 | Axial | 2728 | 71 | 38 |  | 47 |  |

*Max temperature at the center of the AOT (Area of Treatment)

**Supplemental Table 2: Patient 001 Treatment Outcome**

|  |  | Baseline | 2 weeks FU | 1 month FU | 3 months FU | 6 months FU |
| --- | --- | --- | --- | --- | --- | --- |
| **Average Pain (0-10)** |  | **5** | **7.7** | **5** | **3.3** | **5.6** |
| Minimal Pain (0-10) |  | 2 | 5 | 3.3 | 2 | 4 |
| Maximal Pain (0-10) |  | 7 | 9 | 6.6 | 5 | 8.6 |
| SF-MPQ score (0-45) |  | 20 | 19 | 17 | 8 | 17 |
| EQ-5D score (0-15) |  | 9 | 8 | 8 | 6 | 8 |
| PSQI score (0-21) |  | 13 | 16 | 16 | 10 | 14 |
| BPI score (0-70) |  | 39 | 36 | - | 14 | 28 |

**Supplemental Table 3: Patient 005 Treatment Outcome**

|  | Baseline | 2 weeks FU | 1 month FU | 3 months FU | 6 months FU |
| --- | --- | --- | --- | --- | --- |
| Average Pain (0-10) | 6.6 | - | 4.3 | 5.6 | 6.0 |
| Minimal Pain (0-10) | 7.3 | - | 3.3 | 5.3 | 4.6 |
| Maximal Pain (0-10) | 8.0 | - | 5.3 | 6.6 | 6.0 |
| SF-MPQ score (0-45) | 35 | - | 26 | 24 | 27 |
| EQ-5D score (0-15) | 10 | - | 10 | 11 | 10 |
| PSQI score (0-21) | 17 | - | 16 | 19 | 17 |
| BPI score (0-70) | 63 | - | 39 | 38 | 46 |

**Supplemental Table 4: Patient 010 Treatment Outcome**

|  | Baseline | 2 weeks FU | 1 month FU | 3 months FU | 6 months FU |
| --- | --- | --- | --- | --- | --- |
| Average Pain (0-10) | 5.6 | 4.3 | 4.3 | 4.0 | 5.3 |
| Minimal Pain (0-10) | 3.3 | 1.0 | 2.0 | 1.0 | 1.3 |
| Maximal Pain (0-10) | 8.3 | 5.0 | 4.3 | 5.3 | 7.0 |
| SF-MPQ score (0-45) | 16 | 5 | - | 7 | 5 |
| EQ-5D score (0-15) | 9 | 6 | 6 | 6 | 6 |
| PSQI score (0-21) | 12 | 6 | 3 | 9 | 8 |
| BPI score (0-70) | 21 | 16 | 28 | 14 | 19 |

**Supplemental Table 5: Patient 012 Treatment Outcome**

|  | Baseline | 2 weeks FU | 1 month FU | 3 months FU | 6 months FU |
| --- | --- | --- | --- | --- | --- |
| Average Pain (0-10) | 7 | 8 | - | - | - |
| Minimal Pain (0-10) | 0 | 6 | - | - | - |
| Maximal Pain (0-10) | 10 | 10 | - | - | - |
| SF-MPQ score (0-45) | 36 | 32 | - | - | - |
| EQ-5D score (0-15) | 12 | 12 | - | - | - |
| PSQI score (0-21) | 20 | 20 | - | - | - |
| BPI score (0-70) | 70 | 68 | - | - | - |

**Supplemental Table 6: Patient 018 Treatment Outcome**

|  | Baseline | 2 weeks FU | 1 month FU | 3 months FU | 6 months FU |
| --- | --- | --- | --- | --- | --- |
| Average Pain (0-10) | 10.0 | 9.3 | 3.0 | 1.0 | - |
| Minimal Pain (0-10) | 0 | 9.3 | 2.0 | 1.0 | - |
| Maximal Pain (0-10) | 10.0 | 9.3 | 3.0 | 1.0 | - |
| SF-MPQ score (0-45) | 24 | 18 | 4 | 2 | - |
| EQ-5D score (0-15) | 8 | 9 | 8 | 0 | - |
| PSQI score (0-21) | 7 | 5 | 6 | 6 | - |
| BPI (0-70) | 39 | 24 | 33 | 4 | - |

**Supplemental Table 7: MRI findings of the neuromas before and after treatment.**

| **Patient 001 - Before Treatment** | | | |
| --- | --- | --- | --- |
|  | Tibial nerve neuroma | Sural nerve neuroma | Peroneal nerve neuroma |
| Signal | Homogeneous low signal in T1W, heterogeneous intermediate signal in T2W Fat Sat images | Homogeneous low signal in T1W, heterogeneous intermediate signal in T2W Fat Sat images | Homogeneous low signal in T1W, heterogeneous intermediate signal in T2W Fat Sat images |
| Enhancement with Gadolinium | No | No | No |
| **Patient 001 - After Treatment** | | | |
| Neuroma | Tibial nerve neuroma | Sural nerve neuroma | Peroneal nerve neuroma |
| Signal | Homogeneous low signal in T1W images, heterogeneous high signal in T2W Fat Sat images | homogeneous low signal in T1W images, heterogeneous high signal in T2W Fat Sat images | homogeneous low signal in T1W images, heterogeneous high signal in T2W Fat Sat images |
| Enhancement with Gadolinium | Mild | Very mild | Mild |
| **Patient 005 - Before Treatment** | | | |
|  | Tibial nerve neuroma | | |
| Signal | Homogeneous low signal in T1W images, high signal in T2W Fat Sat images | | |
| Enhancement with Gadolinium | Yes | | |
| **Patient 005 - After Treatment** | | | |
|  | Tibial nerve neuroma | | |
| Signal | Homogeneous low signal in T1W images, high signal in T2W Fat Sat images | | |
| Enhancement with Gadolinium | Yes | | |
| **Patient 010 - Before Treatment** | | | |
|  | Tibial nerve neuroma | | |
| Signal | Homogeneous low signal in T1W images, heterogeneous intermediate signal in T2W Fat Sat images | | |
| Enhancement with Gadolinium | No | | |
| **Patient 010 - After Treatment** | | | |
|  | Tibial nerve neuroma | | |
| Signal | homogeneous low signal in T1W images, heterogeneous intermediate signal in T2W Fat Sat images | | |
| Enhancement with Gadolinium | No | | |
| **Patient 012 - Before Treatment** | | | |
|  | Sciatic nerve neuroma | | |
| Signal | Homogeneous low signal in T1W images, high signal in T2W Fat Sat images | | |
| Enhancement with Gadolinium | Yes | | |
| **Patient 012 - After Treatment** | | | |
|  | Sciatic nerve neuroma | | |
| Signal | Homogeneous low signal in T1W images, high signal in T2W Fat Sat images | | |
| Enhancement with Gadolinium | Yes | | |
| **Patient 018 - Before Treatment** | | | |
|  | Sciatic nerve neuroma | | |
| Signal | Homogeneous low signal in T1W images, heterogeneous intermediate signal in T2W Fat Sat images | | |
| Enhancement with Gadolinium | No | | |
| **Patient 018 - After Treatment** | | | |
|  | Sciatic nerve neuroma | | |
| Signal | Homogeneous low signal in T1W images, heterogeneous intermediate signal in T2W Fat Sat images | | |
| Enhancement with Gadolinium | No | | |
